# Dynamical crossover line in supercritical water

**DOI:** 10.1038/srep14234

**Published:** 2015-09-16

**Authors:** Yu. D. Fomin, V. N. Ryzhov, E. N. Tsiok, V. V. Brazhkin

**Affiliations:** 1 Institute for High Pressure Physics, Russian Academy of Sciences, Troitsk 142190, Moscow, Russia; 2 Moscow Institute of Physics and Technology, Dolgoprudny, Moscow Region 141700, Russia

## Abstract

Dynamical crossover in water is studied by means of computer simulation. The crossover temperature is calculated from the behavior of velocity autocorrelation functions. The results are compared with experimental data. It is shown that the qualitative behavior of the dynamical crossover line is similar to the melting curve behavior. Importantly, the crossover line belongs to experimentally achievable (*P*, *T*) region which stimulates the experimental investigation in this field.

In recent decades wide attention of researchers is attracted to the field of supercritical fluids. Supercritical fluids are of extreme importance both for fundamental research and for practical applications especially in chemical industry. In this respect it is important to give an unambiguous definition of the supercritical state itself. For some years a so called Widom line, which is a line of supercritical maxima of correlation length and thermodynamic response functions (isobaric heat capacity *c*_*P*_, isothermal compressibility *k*_*T*_, heat expansion coefficion *α*_*P*_) in fluids, was used to extend a liquid-gas coexistence line into supercritical region^1^. Later on it was shown that Widom line rapidly splits into a bunch of lines of maxima of different quantities as soon as one goes several dozens percent away from the critical point^2^,^3^. Nevertheless the concept of Widom line is quite important for the description of the anomalous behavior near critical point. Several authors reported Widom lines of different systems and arrived to the same conclusion^4^,^5^,^6^,^7^. One can clearly see that another way of demarcation of gaslike and liquid-like fluids beyond the critical point was necessary.

Such a way was proposed in our previous publications^8^,^9^,^10^. These publications introduce a so called Frenkel line which is the line of dynamical crossover in fluids. It was shown that below the Frenkel line the particles of fluid make few oscillations at some quasi-equilibrium position following a jump to another quasi-equilibrium point. This model was proposed by J. Frenkel^11^ after whom the line was named. Above the Frenkel line the particles of fluid move like in a gas, by long jumps before a collision with another particle occurs. As a result below the Frenkel line liquid properties demonstrate some solidlike behavior whilst above it the properties of liquid are similar to a dense gas ones. Impact of crossover from crystallike microscopic dynamics of fluid particles to a gaslike one on different properties of the fluid was addressed in details in refs 8,9. In ref. 10 it was shown that the most convenient way to find the location of Frenkel line in the phase diagram is by monitoring a velocity autocorrelation function (vacf) of the fluid. Basing on this criterion Frenkel line of several model systems (Lennard-Jones and soft spheres^8^,^9^,^10^) and realistic ones (liquid iron^12^, carbon dioxide^7^,^13^, *TIP*4*P*/2005 model of water^13^, methane^13^ and hydrogen^14^) was calculated. This work extends the investigations in the field to the most important liquid - water.

Phase diagram of water is extremely complex. It contains numerous solid phases including both crystalline and amorphous ones. However, the fluid part of the phase diagram is no less interesting. In addition to usual liquid - vapor transition it is widely assumed that a liquid-liquid phase transition (LLPT) takes place in unachievable region of (P, T) parameters^15^. Water demonstrates a set of liquid state anomalies such as density anomaly, diffusion anomaly, structural anomaly and many others^16^. Moreover, water has multiple Widom lines^17^. The first Widom line is related to liqud-gas transition while the second one can be attributed to the hypothetic LLPT. Both Widom lines of water were vividly discussed in literature (see, for example^6^,^18^,^19^,^20^,^21^,^22^, and references therein). In particular, in ref. 21 a connection between Widom line and dynamical properties of water was proposed. Since Frenkel line is the line of dynamical crossover in fluids some kind of relation between the Widom line and Frenkel line can exist.

As it was proposed in our earlier publications several methods to find the location of Frenkel line can be used^8^,^9^,^10^. The most convenient one is based on the lose of oscillations of vacfs. This criterion is used in the present work. Fig. 1(a,b) show the vacfs of oxygens for two densities: *ρ* = 1.0 and 1.3 *g*/*cm*^3^. One can see that the low temperature vacfs for these two densities look qualitatively different while at high temperatures they become very similar. In particular, as the temperature increases the oscillations of vacfs become less pronounced and finally disappear.

One more way to estimate the location of Frenkel line in *P* − *T* or *ρ* − *T* diagram is related to heat capacity of liquid^8^,^9^,^10^. In case of a monatomic fluid the magnitude of *c*_*V*_ at the Frenkel line is 2 *k*_*B*_ per particle (*k*_*B*_ is Boltzmann constant). In case of water the heat capacity *c*_*V*_ undergoes strong decay upon isochoric heating. Next to the melting line the heat capacity per molecule is about 9 *k*_*B*_ or 3 *k*_*B*_ per atom while at high temperatures it becomes as small as 1.5 *k*_*B*_ per atom. If all degrees of freedom of the molecules are excited then the heat capacity per molecule at Frenkel line should be 6 *k*_*B*_ per molecule or 2 *k*_*B*_ per atom. The location of Frenkel line by *c*_*V*_ criterion was evaluated from experimental data. The data were taken from NIST database^23^.

Frenkel line of water obtained from vacf criterion is shown in Fig. 2(a,b). From Fig. 2(a) one can see that Frenkel line starts at the boiling curve at temperature 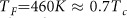, where *T*_*c*_ is the critical temperature. In our previous publications it was shown that the same ratio *T*_*F*_/*T*_*c*_ takes place in Lennard-Jones fluid and liquid iron.

One observes extremely fast grow of the Frenkel line temperature in a narrow interval of densities *ρ* = (1.2–1.22)*g*/*cm*^3^. It is related to extremely slow disappearance of vacf oscillations in this region.

Figure 2(b) shows the location of Frenkel line of water in *P* − *T* phase diagram. Phase diagram of water is very complex. It demonstrates numerous solid phases. In particular, usual ice I melting line has a negative slope. Other solid phases melting lines have positive slopes. The shape of the Frenkel line of water qualitatively resembles the shape of the melting line. At low pressures Frenkel line very slowly increases with pressure. Later on at pressure about 10 *kbar* the slope of the Frenkel line like the one of the melting curve rapidly increases.

Importantly, the relation between the temperature at the Frenkel line *T*_*F*_ and the melting temperature *T*_*m*_ changes upon increasing the pressure. At low pressures (before the rapid increase) the ratio *T*_*F*_/*T*_*m*_ is close to 2. At pressures 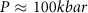 this ratio increases up to 5. On further rise of pressure it reaches the value of 9 at 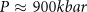. It means that in the range of pressures considered in the present work the Frenkel line bends up with respect to the melting line.

In our previous publications it was proposed that in the limit of high pressures Frenkel line should be parallel to the melting line in double logarithmic coordinates. In case of water we are not aware of any measurements of the melting curve above 1000 *kbar*. We expect that the Frenkel line and the melting curve will be parallel in the high pressure limit, but one needs to extend the melting curve to higher pressures in order to check it. Although this conclusion may be violated by transition of water into superionic phase under high pressure^24^. This phenomena can not be taken into account in frames of purely classical model used in the present work.

Very recently Frenkel line of water calculated by vacf criterion for a different model (*TIP*4*P*/2005) was reported^13^. This line is shown in Fig. 2(b) for the sake of comparison. One can see that this line is systematically higher then our line. However, the lines are very close to each other and this small discrepancy can be attributed to the different models under investigation. The authors of^13^ studied the Frenkel line of water up to *P*_*max*_ = 30 *kbar*. In our work the Frenkel line is traced up to pressures as high as almost *P*_*max*_ = 2000 *kbar* where allowed us to see an interesting phenomenon. From Fig. 2(b) one can see that the bend of Frenkel line in double logarithmic coordinates takes place at pressure about 50 *kbar*. One can relate this bend to some changes in the local structure of the liquid.

Figure 2(b,c) show also the location of Widom line of *TIP*4*P*/2005 model of water calculated from maxima of isobaric heat capacity *c*_*P*_ in ref. 21. As it was shown in several recent publications the supercritical maxima of different substances rapidly vanish on departing from the critical point^2^,^3^,^4^,^5^,^6^,^7^,^25^. In ref. 21 the Widom line extends up to the pressure 0.38 *kbar* which is lower then the Frenkel line starts. Moreover, extrapolation of the Widom line to higher pressures should go above the Frenkel line of both *SPC*/*E* and *TIP*4*P*/2005 models of water. From this one can conclude that these lines are not related to each other. One can assume that at low pressure the crossover of dynamical properties of liquid is governed by the Widom line while at higher pressures it is determined by the Frenkel line.

Let us consider the coordination number of oxygens. The coordination number can be calculated from radial distribution function (rdf): 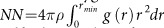, where *ρ* is the number density and *r*_*min*_ is the location of the first minimum of rdf. The number of nearest neighbors (NN) along the isotherm *T* = 1000 *K* is shown in Fig. 3. NN of water along the melting line was reported in refs 26,27. Although our results belong to an isotherm while the results of these publications are related to the melting line, the NNs are calculated in similar pressure interval. That is why we show them in the same plot for comparison. The difference between this work and the literature data should be referred not only to different lines in *P* − *T* plane but also to different models studied and different methods of calculation. One can see that up to pressure of approximately 50 *kbar* the coordination number rapidly increases while above this threshold it holds approximately constant. One can conclude, that at small pressures the local structure is very sensitive to the pressure change while at higher ones the local structure is very stable which is similar to the case of simple liquid. Therefore one can say that water becomes “simpler” upon increasing pressure.

We expect that similar rapid increase of Frenkel line in the density range 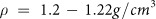 can be observed in *TIP*4*P*/2005 as well. However, the results reported in the work^13^ end up at the density 1.2 *g*/*cm*^3^. So one needs to extend them in order to check this assumption.

We report a computational study of dynamical crossover in water. The temperature of crossover (the Frenkel line temperature *T*_*F*_) is calculated from the behavior of velocity autocorrelation functions. The results are compared to the experimental ones obtained from isochoric heat capacities. It is shown that qualitative behavior of Frenkel line is similar to the behavior of the melting curve. However, the ratio *T*_*F*_/*T*_*m*_ increases with increasing of pressure. It means that the lines diverge in the range of pressures considered in the present work. This divergence can be related to the change of the local structure of water upon increasing the pressure.

Importantly, the temperatures of dynamical crossover appear to be rather low at moderate pressures. At pressures *P* < 30 kbar *T*_*F*_ does not exceed 1000 K. It means that (*P, T*) parameters of Frenkel line can be achieved experimentally. Such experimental works would be important not only for deeper understanding of dynamical behavior of liquids but also could serve for supercritical technology. Importantly, the properties of fluids at the Frenkel line are close to the optimal ones for technological applications^28^. Water as well as carbon dioxide are among the most important and widely used supercritical fluids^29^. Supercritical water is used for many different applications, such as green solvent, as reaction medium for different chemical processes, for production of biofuel, oxidation of hazardous materials which is important for dangerous waste disposal. Applications of supercritical water include separation, extraction and purification of different substances and many others^30^.

The principal property of supercritical water providing its widespread application is its solving power. In ref. 13 the solubility of different solutes in carbon dioxide and its relation to the Frenkel line was discussed. It was shown that the solubility maxima are close to the Frenkel line. However, the data for solubility maxima of different solute in supercritical water are not available at the moment. Basing on the discussion of^13^ and the results of the present paper one can propose that the optimal solving power of water should belong to the interval *T* = 700–1000 *K* and *P* = 1–3 *GPa*. Currently, the most widespread usage of supercritical water belongs to the temperatures interval 650–1000 *K* and to the pressures up to 0.5 *kbar*. However, these (*P, T*) conditions were found empirically and do not have any solid theoretical ground. Moreover, up to now the supercritical technology advances the theoretical foundations in the field. This work as well as ref. 13 allow to predict the best (*P, T*) conditions for supercritical water application which makes these publications the pioneering works in developing the theoretical basis of supercritical technologies.

## Methods

In the present work we study the behavior of water by means of molecular dynamics simulations. An *SPC*/*E* model of water is used^31^. The phase diagram of this model was reported in several publications. In^32^ a comparison of solid part of the phase diagram of *SPC*/*E*, several variants of *TIP*4*P* model and experimental results is given. One can see that all models fail to reproduce the whole complexity of the experimental phase diagram, but manage to describe some parts of it. In particular, *SPC*/*E* model is good in describing boiling curve of water. In ref. 33 boiling curve of *SPC*/*E* water is reported. The critical parameters are found to be 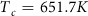, 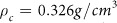 and 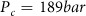. Experimental critical point of water corresponds to 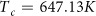, 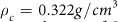 and 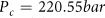. One can see that except some difference in critical pressure the critical point of *SPC*/*E* model is very close to the experimental one. Moreover, in ref. 34 a comparison of *SPC*/*E* model and ab-initio results at high pressures and high temperatures was reported. It was shown that the discrepancy of *SPC*/*E* model and ab-initio results is of the order of 15–20% for *T* = 1000 *K* and pressure up to about 100 *kbar*. However, at *T* = 2000 *K* and pressures up to approximately 90 *kbar* the agreement of ab-initio and *SPC*/*E* results is within 5% which should be considered as good agreement. One can guess that at *T* = 1000 *K* and so high pressure the results are affected by crystallization effects while at temperatures well above the melting line *SPC*/*E* model can be used to study the high pressure behavior of water.

A system of 4000 water molecules in a cubic box was simulated in molecular dynamics at constant volume, number of particles and temperature (canonical ensemble). The temperature was held constant by Nose-Hoover thermostat. The density was varied from 
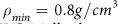
 up to 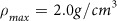 and the temperatures from *T*_min_ = 275 *K* up to 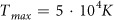. Initially the system was equilibrated for 1*ns* with a time step *dt* = 1*fs*. After that it was simulated more 1*ps* with the same time step in order to calculate the thermodynamic properties. Finally, 10^5^ steps with timestep *dt* = 0.1*fs* were made in order to well reproduce the decay of velocity autocorrelation function (vacf).

All simulations were performed using lammps simulation package^35^.

## Additional Information

**How to cite this article**: Fomin, Y. D. *et al.* Dynamical crossover line in supercritical water. *Sci. Rep.* **5**, 14234; doi: 10.1038/srep14234 (2015).

## Figures and Tables

**Figure 1:**
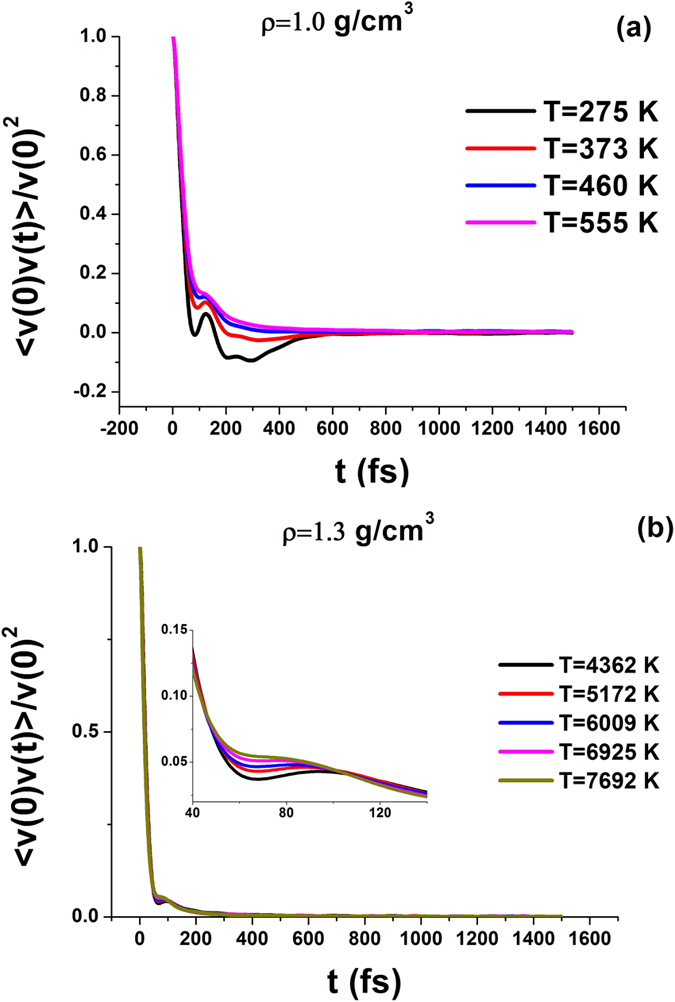
Normalized velocity autocorrelation functions of oxygen atoms at density (a) *ρ* = 1.0 *g*/*cm*^3^ and (b) *ρ* = 1.3 *g*/*cm*^3^. The inset in panel (**b**) enlarges the time period 40–140 ps where the oscillation behavior of vacfs takes place.

**Figure 2:**
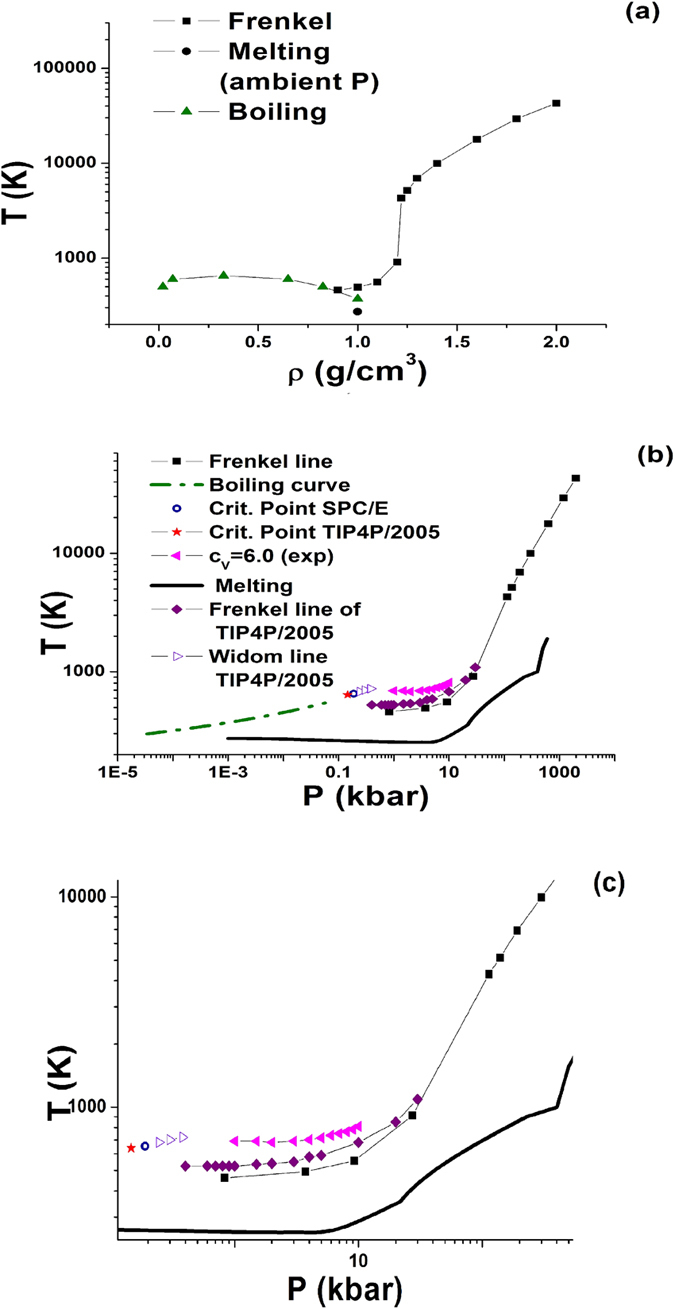
Location of Frenkel line of water in the phase diagram in (a) *ρ* − *T* and (b) *P* − *T* coordinates. The Frenkel line is obtained from vacf criterion. In case of *P* − *T* diagram the boiling curve corresponds to the experimental curve^36^, the critical point is taken for SPC/E model^33^. The melting line is combined from several publications^24^,^37^,^38^,^39^,^40^,^41^,^42^. Experimental data for heat capacity are taken from NIST database^23^. The Frenkel line of *TIP*4*P*/2005 water is taken from ref. 13. Widom line of *TIP*4*P*/2005 model is taken from ref. 21. Panel (**c**) enlarges the moderate pressure part of the diagram. The notation of this panel is the same that in panel (**b**).

**Figure 3:**
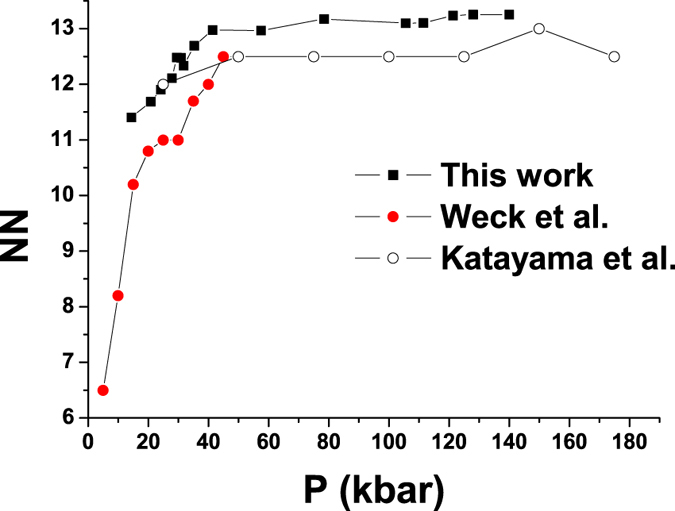
Number of nearest neighbors of oxygen along *T* = 1000 *K* isotherm. For comparison literature data of NN along the melting line are also given. The data are taken from ref. 26 (Weck) and ref. 27 (Katayama).
